# Development of a three-dimensional model of the human respiratory system for dosimetric use

**DOI:** 10.1186/1742-4682-10-28

**Published:** 2013-05-01

**Authors:** Jacky A Rosati Rowe, Ray Burton, George McGregor, Rob McCauley, Wei Tang, Richard Spencer

**Affiliations:** 1 US EPA Office of Research and Development, Durham, NC 27711, USA; 2 Lockheed Martin, 109 TW Alexander Drive, Research Triangle Park, NC 27711, USA

**Keywords:** Three-dimensional modeling, Lung physiology, Nasal physiology, Dosimetry, Human respiratory system, Sensitive populations

## Abstract

**Background:**

Determining the fate of inhaled contaminants in the human respiratory system has challenged scientists for years**.** Human and animal studies have provided some data, but there is a paucity of data for toxic contaminants and sensitive populations (such as children, elderly, diseased).

**Methods:**

Three-dimensional modeling programs and publicly available human physiology data have been used to develop a comprehensive model of the human respiratory system.

**Results:**

The *in silico* human respiratory system model, which includes the extrathoracic region (nasal, oral, pharyngeal, and laryngeal passages), the upper airways (trachea and main bronchi), the tracheobronchial tree, and branching networks through alveolar region, allows for virtually any variation of airway geometries and disease states. The model allows for parameterization of variables that define the subject’s airways by integrating morphological changes created by disease, age, etc. with a dynamic morphology.

**Conclusions:**

The model can be used for studies of sensitive populations and the homeland security community, in cases where inhalation studies on humans cannot be conducted with toxic contaminants of interest.

## Introduction

Morphologically realistic models of human organ systems have become critical to research advancement in a changing health research world of dwindling research funding, the push to phase out animal toxicology studies [1], and the need for immediate answers to protect human health. Research is also challenging in such areas as the homeland security community, where inhalation studies on humans cannot be conducted with contaminants of interest (e.g., *B. anthracis*, ricin, etc.) because of toxicity. An ideal solution is thus provided by a morphologically realistic computational model of the human respiratory system which can respond to the dynamic changes of respiratory mechanics and abnormal pathologies.

Precursors to this morphologically realistic computer model included numerous mathematical and flow models that looked at the deposition of particles in the human lung [2]. There have also been a few nasal and oropharyngeal models investigating the movement of particles in the nasal passages and mouth/pharynx region [2,3]. Research has even recently advanced to produce a three-dimensional model from computed tomography data that included a simulated oral cavity, pharynx and larynx, and seven generations into the airways [4]. The latter model used computational fluid dynamics (CFD), including large-eddy simulations, to determine regional particle deposition, but was limited due to its lack of morphological realism, e.g., the lack of nasal passages, oral passage with tongue and teeth, uvula, five lobe lung, 23 branching generations, etc.). Recent work by Corley et al. [5] has developed a 3-D CFD airway model that extends from the external nares or mouth to the bronchiolar region of the human lung based on CT imaging of the head and torso of a female volunteer, although the airway only extends to the 9th generation due to the limitation posed by the resolution of the CT scanner.

Until now, no model has combined a physiologically based nose, larynx, pharynx, mouth, and lungs to create a three-dimensional morphologically realistic model of the human respiratory tract from the nares to the alveoli. The current model can be used to simulate inhalation, deposition, and exhalation of contaminants, and is progressing towards the consideration of age, race, gender, and health. This virtual human respiratory system includes the extrathoracic (ET) region (nasal, oral, pharyngeal, and laryngeal passages), the upper airways (trachea and main bronchi), the trachea bronchial tree, and branching networks down through alveolar region.

## Methods

The current morphology modeling of the upper airway system began with the Visible Human Project’s (VHP) Brigham and Women’s Hospital (BWH) head image data. The acquisition of transverse MRI and Computed Tomography (CT) image data of the head of a 72-year-old male subject, with cryosections at 0.174 mm intervals and photographed at a resolution of 1056 × 1528 pixels, was performed at BWH, Harvard Medical School, under contract to the National Library of Medicine (NLM). This dataset is available to Virtual Human Project license holders and the images can be found in the directory BWH_Harvard when logged on to the NLM image server.

**Figure 1:**
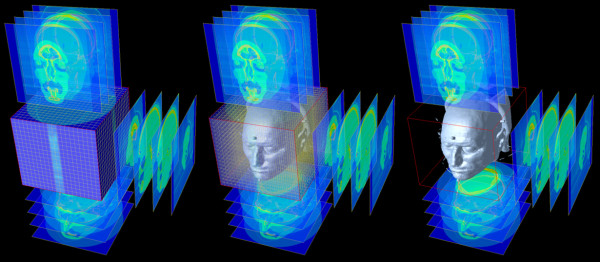
Construction of a volumetric dataset from a series of image slices and initial three-dimensional representation of the underlying structure of a human head.

The exterior features of the human head, the skull, and details of nasal and oral passages were reconstructed from a coronal series of 1,528 cryosection images, with each image having a resolution of 1036 × 1477 pixels. The images were stacked and combined into a single volumetric dataset. Each voxel or volumetric element represents a value in a three-dimensional, regular grid with 2,338,102,816 elements (Figure 1).

**Figure 2:**
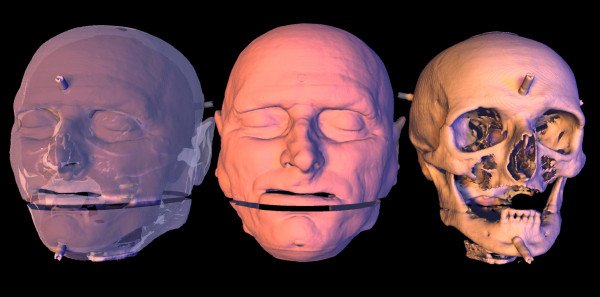
The extracted three-dimensional representation of the underlying soft tissue structure of the human head and the hard bone structure of the skull.

Using an internal software implementation of a marching cubes algorithm [6], several threshold values were used to extract a three-dimensional surface, or isosurface, that represents all the points in the volume of a constant value or threshold value. This technique is analogous to a three-dimensional contour. Bone structures such as the skull and soft tissue features, including the facial features, were extracted (Figure 2).

### Extrathoracic morphology development

The structure of the nasal and oral cavities, pharynx, epiglottis, larynx, and esophagus of this subject were created separately using the marching cubes algorithm [6] and extracting threshold isosurfaces.

#### Nasal morphology development

The nasal cavity is composed of two narrow passages that are separated by the nasal septum. Each nasal passage features three curved fin-like airway protrusions known as the superior, middle, and inferior meatus. Extending further into the airways, the two passages merge at the distal end of the nasal cavity and form the beginning of the nasopharynx. The floor of the nasal cavity is formed by the hard palate, which is also the roof of the mouth.

**Figure 3:**
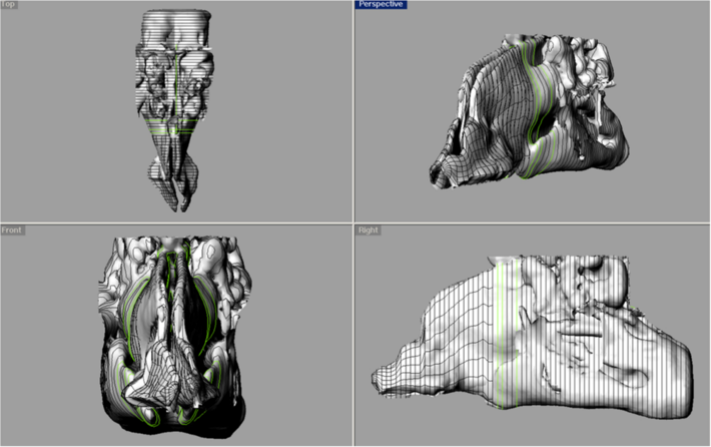
**The nasal cavity is shown as the composition of a series of curves obtained from the BWH head data.** Parts of the nasal cavity such as the sinuses were removed from the model to match structures from published literature.

**Figure 4:**
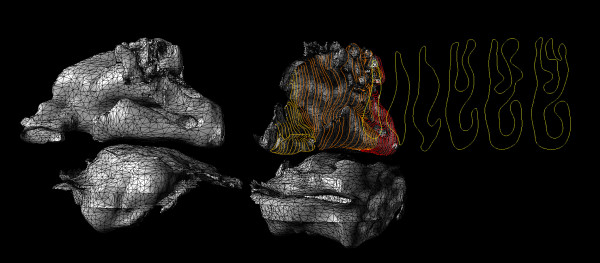
**Contour lines or profile curves were extracted from the nasal cavity mesh obtained via the marching cubes algorithm.** These curves are necessary to maintain precise structural cohesion to the original mesh and uphold the underlying structure of the concha (or turbinate) found in all nasal structures.

The nasal cavity region was extracted from the volume data and isolated using Rhinoceros (also referred to as Rhino3D – a Non-Uniform Rational B-Spline (NURBS) modeling package) developed by Robert McNeel & Associates (Seattle, WA) (Figure 3). Parts of the nasal cavity such as the sinuses were removed from the model to match structures from published literature. Using the nasal isosurface representation extracted from the volumetric data as a reference surface mesh, an evenly spaced series of planar curves and points were created from the intersection of a cutting plane through the mesh surface. These contour lines or profile curves were used to fit a new surface through selected profile curves that define the surface shape (Figure 4).

**Figure 5:**
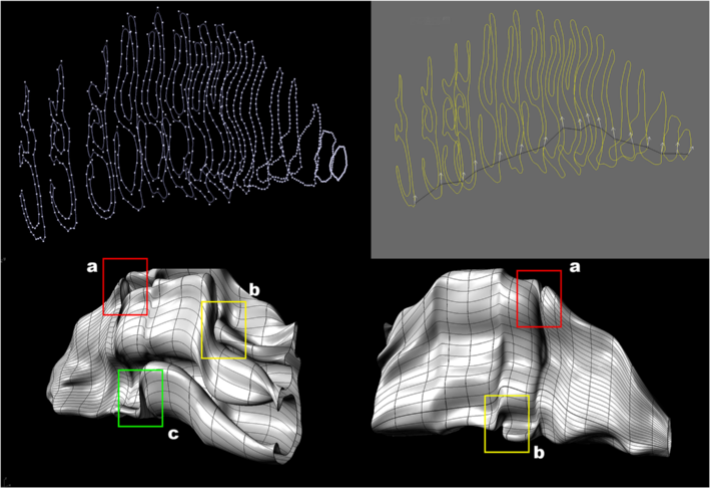
Anomalies in the nasal surface mesh occurred such as (a) twisting in the surface (b), intersecting surfaces, and (c) pinching or extruded surfaces.

**Figure 6:**
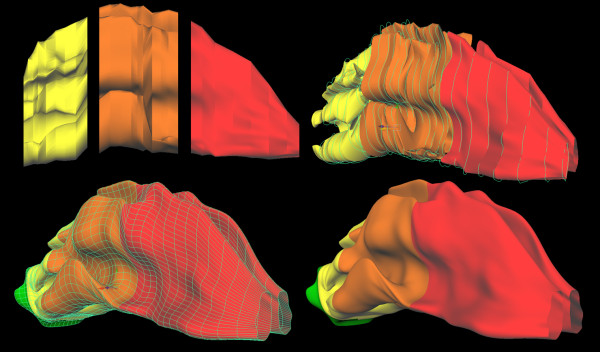
This image illustrates surface segmentation based on profile complex and the final surface mesh of the nasal cavity free from surface anomalies.

Anomalies in the nasal surface mesh occurred such as (a) twisting in the surface (b), overlapping or intersecting surfaces, and (c) pinching or extruded surfaces. These anomalies occurred during the initial surface reconstruction because of the complex shape and varying degree of the turbinates and because of the arbitrary number of vertices of the contour lines did not have the same curve degree and edit points which made the auto surfacing lofting function fail (Figure 5). These anomalies were corrected by auto lofting profile curves with the same curve degree and the same number of edit points into surface segments (Figure 6).

The final stitching of the nasal passage surface segments was completed by rebuilding the profile curves, hand manipulating the curve degree and number of edit points on adjacent surfaces segments, and essentially combining two shapes or segments creating a surface between them manually lofting the surfaces to form a closed watertight topology free from intersecting and twisted surfaces (Figure 6).

The resulting model was free of surface defects and was sufficiently smooth such that the resulting model is accurate to the original isosurface mesh obtained from the marching cubes algorithm [6]. To combine the models effectively, we use merging, sewing, and hole-filling tools that provide flexibility in the joining of the surface segments.

#### Oral cavity morphology development

**Figure 7:**
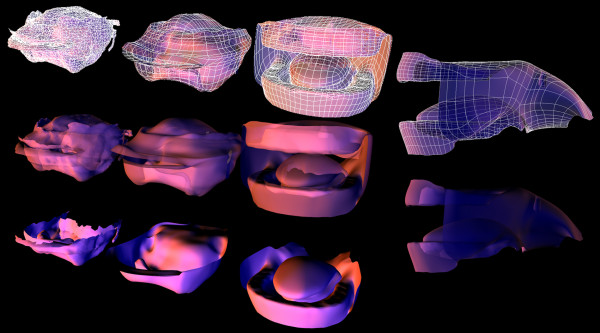
The oral cavity model topology, or surface structure, was simplified to produce a more suitable structure.

The need to understand and predict airflow patterns through the oral cavity required creating a physically realistic morphology of both the tongue and oral cavity. The oral cavity extracted from the volumetric data was insufficient to be used as a morphologically realistic model (Figure 7). A Digimation dental model VP1298 (Lake Mary, FL) was used as a reference mesh for the teeth with gums. The two separate parts — the upper and lower gums and teeth — were combined to form a complete oral cavity. The tongue and the entire oral cavity were retopologized. The tongue was added to the oral model. The upper airway models were then aligned and combined into a single morphology model, including sides of the oral cavity and uvula.

### Combining the extrathoracic segments

To create a watertight extrathoracic model suitable for CFD studies, additional surface anomalies were corrected for all segments. These included non-manifold surfaces; different details, structures, and resolution of surfaces; matching surfaces; holes in the mesh at critical junction points; and other issues that occurred throughout the stages of combining the separate parts into a functional and morphologically-realistic model. A non manifold surface occurs when more than two surface elements (triangles) intersect on one edge.

**Figure 8:**
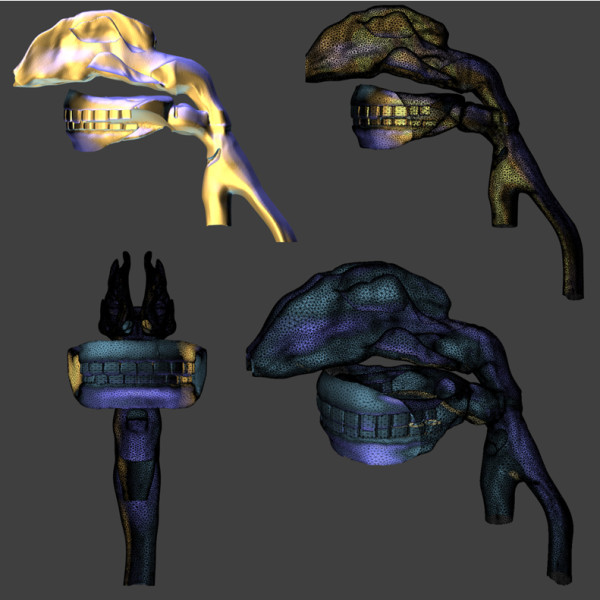
Views of the extrathoracic airways combined with new oral cavity structures detail, complete with teeth, gums, tongue, uvula, and epiglottis.

A triangulated mesh was created with manifold surfaces that could be imported directly into computational engineering software for further analysis. A combined model required the use of merging, sewing, and hole-filling tools (provided in the commercial application Rapidform XOR), which allowed for flexibility in joining the separate models (Figure 8).

### Airway development

To develop a robust method of generating lung morphologies for CFD studies, we have integrated the most promising aspects from numerous modeling techniques. The dynamic surface modeling technique we have developed uses data from idealized models to construct three-dimensional computer simulations of tubular airway structures within lungs. These anatomically accurate computer representations of human lungs are in a format such that their graphical displays may be used in the medical arena, and the resulting airway geometries can be used in CFD analysis. This robust morphology generation system can generate three-dimensional tubular airway structures using surface modeling techniques from existing anatomical data.

To describe the three-dimensional structure within lungs, each airway in the branching network must be uniquely defined in terms of its spatial coordinates. Therefore, each airway is represented by its parameterization into length, diameter, and angles of orientation. The orientation angles consist of the bifurcation angle between two branching airways, and the rotation angle, which is a measure of the rotation of the plane containing the branching airways. We use the morphology to describe a symmetric, dichotomously branching system in which a parent airway branches into two daughter airways. This morphology was developed based on work by Weibel [7], Soong [8], Yeh and Schum [9], Phalen et al. [10], and Lovelace Biomedical & Environmental Research Institute [11]. It accounts for the inter-subject variability present in a typical adult population by examining probability distributions of the airway data. It is divided into 24 generations beginning with the trachea (generation 0), and ending at the alveolar sacs (generation 23). A mean value for each airway parameter is assigned for each generation. In our model, the first three generations are asymmetric representing the actual geometry of the trachea and large bronchii. Beyond these generations, the symmetric branching data is used.

The airway lengths and diameters are taken from a typical adult lung. To be anatomically realistic, we have used the branching angles, which are based on previous measurements of morphometric data [7-11], and we assumed a constant rotation angle of 90° throughout the airway network. Simply stated, the morphology is generated automatically from anatomical data, and can be controlled and modified in a scientific context within the bounds of that data.

Generating surface models of airway morphologies using anatomic data involves abstracting the task of creating a complex lung model, containing over 16 million airways, to the knowledge-based parameterization of its constituent airways. The tubular (right circular cylinder) structure of an airway is defined by its length and diameter, with its position defined by the orientation angles. The dichotomously branching system of airways that comprise the lung can be treated as a contiguous series of cylindrical tubes connecting at Y-shaped bifurcations. In creating the surface models of the airways, notable complexities arise in the formations of the bifurcation shapes (i.e., where the airways intersect). In this implementation, the models are complex surfaces composed of high-level NURBS surface patches. These surface patches are then stitched together both along the opposing lengths and around the ends where they connect with their parent or daughter airways. Each surface patch is built from a relatively coarse mesh of control points generated by algorithms using the basic length, diameter, and angle input parameters. The density of these control points is higher at the junction of the bifurcations so that the transition region between airways can be adjusted to be either smoother or sharper than shown.

The surface modeling techniques we have employed to generate the morphologies allowed us to create smooth connecting airways and realistic carinal ridge shapes, thus obtaining anatomically accurate representations of the bifurcating systems. This has been shown to be very important to lung airstreams and particle deposition [12], and were necessary to obtain realistic geometries that could then be further refined and exported to CFD software to study airflow properties and deposition patterns of inhaled aerosols in the lung. The NURBS surface model was used as a reference mesh for polygonal meshes built in Maya.

**Figure 9:**
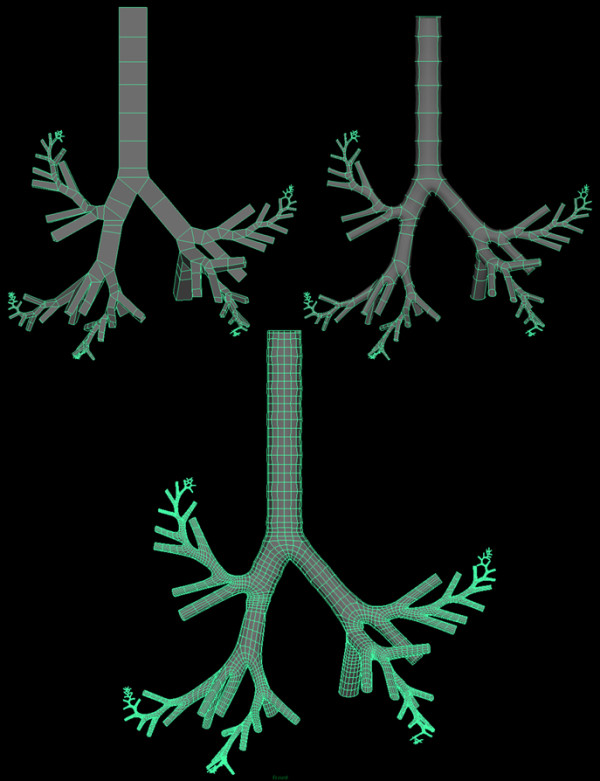
**Bifurcation model below the pharynx showing one complete 24 generation path in each of five lobes using the subdivision surfaces method.** The upper left image shows the original polygon cube model. The upper right image shows the preview caged mode in Maya. The lower image shows the final result of the subdivision surfaces method.

We developed a method of generating a three-dimensional computer morphology model of the human lung branching airway network using Autodesk Maya 3-D software (San Rafael, CA). Maya displays polygonal meshes in a cubic wireframe form. It also provides a quick and simple method to view the mesh in a cage mode view, which is a smoothed version of the mesh with a cubic wireframe cage surrounding the mesh. The final display method provided is a smooth mesh mode. A subdivision surface (subdiv) in the field of three-dimensional computer graphics is a method of representing a smooth surface by specifying a coarser piecewise linear polygon mesh. The smooth surface can be calculated from the coarse mesh as the limit of a recursive process of subdividing each polygonal face into smaller faces that better approximate the smooth surface. The bottom four vertices of a single polygon cube are selected, scaled, and adjusted to form the bottom apex for the initial triangle that forms all bifurcation intersections in the branching network. The top and the two side faces of the cube are extruded and rotated to form the bifurcation intersections. Finally, the bifurcation intersection is displayed in the cage mode view to visualize how the resulting smoothed structure will appear (Figure 9).

The smooth mesh preview steps only changes the display of the mesh; this enables the user to visualize how the mesh will appear when the mesh is smoothed. The original mesh remains in its original cubic form until the final mesh is converted to a fully smoothed equivalent in a subdivision proxy form. This is done using the Smooth Mesh Preview to Polygons tool which converts a Smooth Mesh Preview version of a polygon mesh to an actual polygon mesh. The attribute settings for the Smooth Mesh Preview are used when converting to the polygon mesh.

### Completing the internal structures model

**Figure 10:**
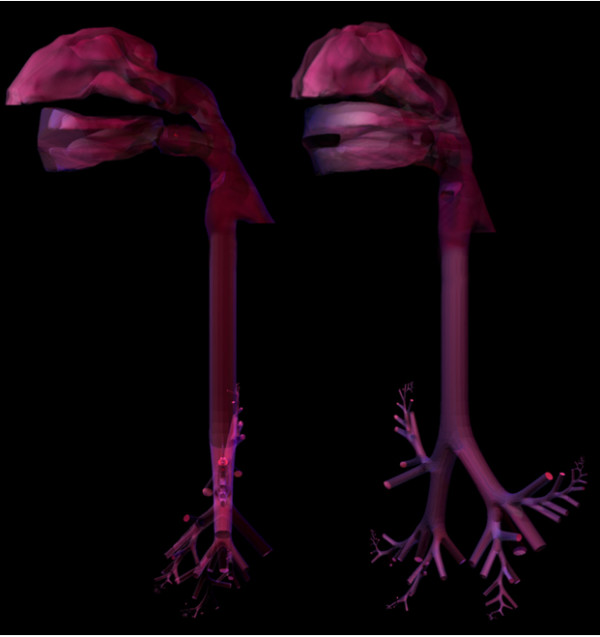
The full 5-lobe model was meshed to be watertight with no surface imperfections.

The internal structures of a physiologically based nose, larynx, pharynx, mouth, and lung bifurcations were combined to create a three-dimensional morphologically realistic model of the human respiratory tract from the nares to the alveoli (Figure 10). The same method was used as described when combining the extrathoracic models. Briefly, a triangulated mesh was created with manifold surfaces that could be imported directly into computational engineering software for further analysis. A combined model required the use of merging, sewing, and hole-filling tools (provided in the commercial application Rapidform XOR), which allowed for flexibility in joining the separate models (Figure 10).

### External structure rebuild

The polygon surface mesh obtained from the marching cubes algorithm [6] resulted in a highly dense mesh of 498577 vertices, 1411868 edges and 921622 faces. As our ultimate goal was to have a deformable mesh, it was desirable to have a less dense mesh that would allow us to morph the model to vary race, gender and age.

**Figure 11:**
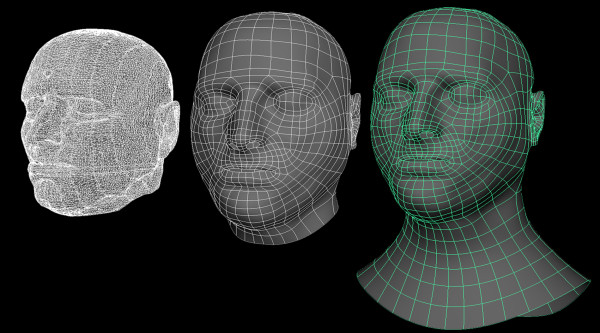
Reconstruction of the surface topology of the soft tissue representing the facial features.

The original triangulated surface mesh topology in which each surface element is made up of triangles is not suitable for deformation. Using a stand-alone resurfacing and maps baking application, TopoGun (PIXELMACHINE SR), the triangulated surface mesh topology was rebuilt to take advantage of a surface constructed with quadrilaterals or quads. The reasoning for using quads as the surface mesh topology element revolves around subdividing, edgeloops, and smoothing. Quads surface elements subdivide evenly, which is better for flexible organic models. A particular flow of vertices in a mesh is called an edgeloop which allows you to control how the surface bends and folds while the mesh is deformed, morphed, or animated. Quad surfaces also provide the basis for adding mesh details such as creating surface folds or wrinkles, adding muscles, and sharpening edges. Edgeloops are a closed loop of vertices and can be followed around a mesh until it returns to its origin. The surface topology soft tissue representing the facial features was reconstructed to move from triangulated mesh to quad surface elements to allow for flexibility and movement during deformation and morphing of the features (Figure 11).

**Figure 12:**
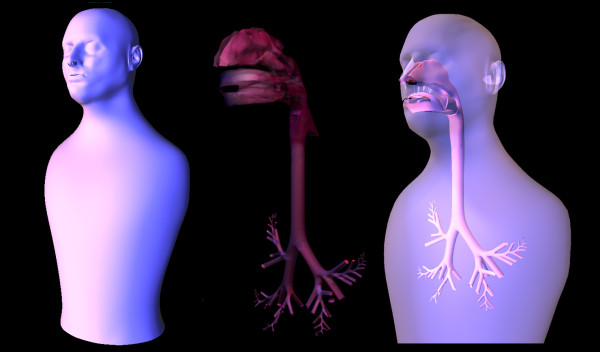
Complete contiguous internal and external typical path model, including nasal and oral, cavities, larynx, and 24 generations with a single bifurcating path into each of the five lobes of the lung.

The new retopologized head mesh was joined with an idealized external torso, and combined with the internal structure from Figure 10. The nasal and oral cavities were attached to the external nose and mouth by aligning like points on the structures. The model, as seen in Figure 12, can respond to the dynamic changes of respiratory mechanics and abnormal pathologies.

### Recent advances

Current development of the model includes the addition of physiologically realistic cartilaginous rings; a parameterized implementation of automatic generation of bifurcations; and the addition of FaceGen (Singular Inversions, Toronto, ON) software to create realistic three-dimensional human faces adjusted for age, race, gender, and other controls, or fit specifically to a photograph. These faces can be applied to the developed polygonal meshes; internal oral and nasal structures can be attached and manipulated based on facial characteristics. These advances are expected to be fully incorporated into the current model by September of 2013. In addition to the current male model, an adult female model and a child/infant model are under development using the same techniques presented in this paper.

### Summary

A state-of-the-art, morphologically realistic model of the human respiratory tract from the nares to the alveoli has been developed that simulates inhalation, deposition, and exhalation of contaminants. It includes the upper respiratory tract, the extrathoracic region (nasal, oral, pharyngeal, and laryngeal passages), the upper airways (trachea and main bronchi), the tracheobronchial tree, and branching networks down through alveolar region.

We are currently parameterizing variables that define the subject’s airways so that the model can integrate morphological changes created by respiratory disease, exposure to toxins or stressors, and age, along with a dynamic morphology that mimics the changes in the structures during a typical breathing cycle. The model will allow for virtually any variation of airway geometries and disease states. Such flexibility will be critical to investigating sensitive populations, such as children, the diseased, and the elderly. The model will also be critical to the homeland security community where inhalation studies on humans cannot be conducted with contaminants of interest (e.g., *B. anthracis*, ricin, etc.) because of toxicity.

The model may be used to predict dose from exposure to hazardous particulate-based contaminants, such as anthrax, ricin or particulate-based hazards. It may also prove useful in targeting pharmaceuticals to the appropriate location in a specific individual’s respiratory system.

## Competing interests

The authors declare that they have no competing interests.

## Authors’ contributions

Dr. JAR, an aerosol scientist with experience in dosimetric research and modeling, is the principal investigator on this work. Dr. R has led the research to develop this model, including model design, scope, and data procurement; she also led the development of the manuscript. Mr. RB, a scientific visualization specialist, procured the data for and performed the modeling of the airways and the extrathoracic regions. Mr. GMcG, scientific applications consultant, assisted with the airway modeling. Mr. RMcC, scientific applications consultant, performed modeling of the human lung and is currently leading the development of a dynamic mesh for the model. Dr. WT, senior modeling engineer, assisted with the airway modeling and flows. Mr. RS, scientific applications consultant, assisted with the original airway model development. All authors read and approved the final manuscript.
